# 1082. Clinical Characteristics and Vertical Transmission of Pregnant Women with SARS-CoV-2 Infection and Their Neonates

**DOI:** 10.1093/ofid/ofac492.923

**Published:** 2022-12-15

**Authors:** Jiyoung Lee, Mi-Young Lee, Euiseok Jung, Ji-Na Lee, SeongMan Bae, Jiwon Jung, Min Jae Kim, Yong Pil Chong, Sang-Oh Lee, Sang-Ho Choi, Yang Soo Kim, Sung-Han Kim

**Affiliations:** Asan medical center, Seoul, Seoul-t'ukpyolsi, Republic of Korea; Asan medical center, Seoul, Seoul-t'ukpyolsi, Republic of Korea; Asan medical center, Seoul, Seoul-t'ukpyolsi, Republic of Korea; Asan medical center, Seoul, Seoul-t'ukpyolsi, Republic of Korea; Asan medical center, Seoul, Seoul-t'ukpyolsi, Republic of Korea; Asan Medical Center, Seoul, Seoul-t'ukpyolsi, Republic of Korea; Asan Medical Center, Seoul, Seoul-t'ukpyolsi, Republic of Korea; Asan Medical Center, Seoul, Seoul-t'ukpyolsi, Republic of Korea; Asan Medical Center, Seoul, Seoul-t'ukpyolsi, Republic of Korea; Asan Medical Center, Seoul, Seoul-t'ukpyolsi, Republic of Korea; Asan Medical Center, Seoul, Seoul-t'ukpyolsi, Republic of Korea; Asan medical center, Seoul, Seoul-t'ukpyolsi, Republic of Korea

## Abstract

**Background:**

Pregnant women with SARS-CoV-2 infection are known to have a poor prognosis. In addition, the previous meta-analysis revealed that SARS-CoV-2 infection in neonates born from pregnant women with SARS-CoV-2 infection is about 2%. However, there are limited data on the clinical characteristics of pregnant women with SARS-CoV-2 infection and their neonates and the vertical transmission rate in South Korea.

**Methods:**

Pregnant women confirmed as SARS-CoV-2 infection were retrospectively reviewed in Asan Medical Center from September 1 2020 to April 26 2022. All neonates from SARS-CoV-2-infected women underwent SARS-CoV-2 PCR within 24 hours after the birth and 48-hour interval if he or she stayed in the hospital.

**Results:**

A total of 60 pregnant women gave birth by cesarean section (n=40, 67%) or vaginal delivery (n=20, 33%). Among them, three women gave birth to twins (63 neonates). Delivery was carried out at the average gestational age of 268 days (± 14.0), and 9 patients (15%) had underlying diseases. Of these 60 patients, 11 (18%) received COVID-19 vaccination. Pneumonia was confirmed by chest radiograph in 7 patients (12%), and 2 patient (3%) required supplemental oxygen therapy who eventually recovered. The mean weight of 63 newborns was 3137 g (± 558), and 8 neonate (13%) was a low-birth weight (< 2500 g), and 12 neonate (19%) was premature (< gestational age 37 weeks). Apgar score was 8.1 points (± 1.2) at 1 minute and 9.1 points (± 0.8) at 5 minutes. Five neonates (8%) required mechanical ventilation, who eventually recovered. All 63 neonates revealed negative SARS-CoV-2 PCR results with 24 hours after the birth. After 48 hours, 45 newborns exhibited negative SARS-CoV-2 PCR results. So, there was no vertical transmission among 63 neonates (0%, 95% CI 0-6).

**Figure:**
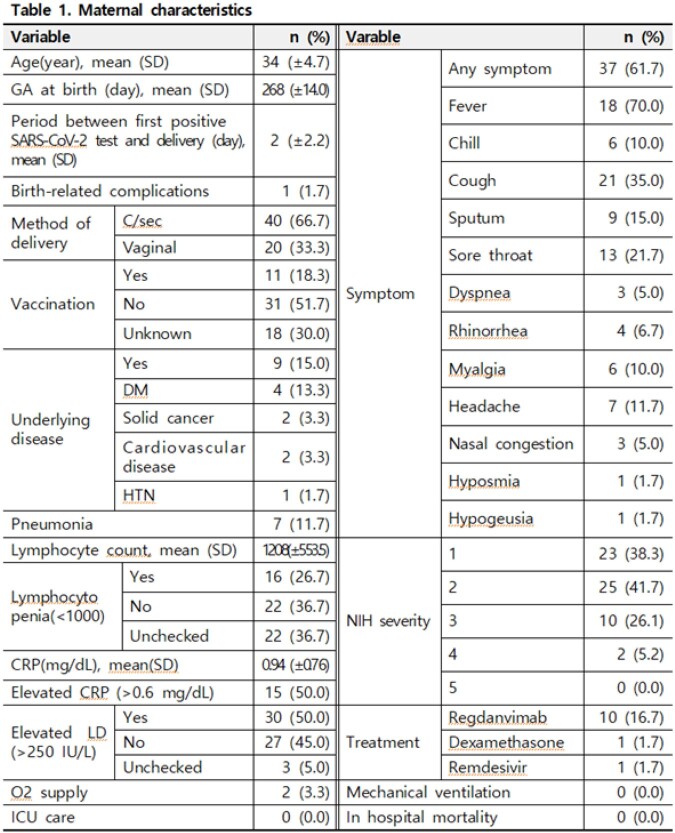

**Figure:**
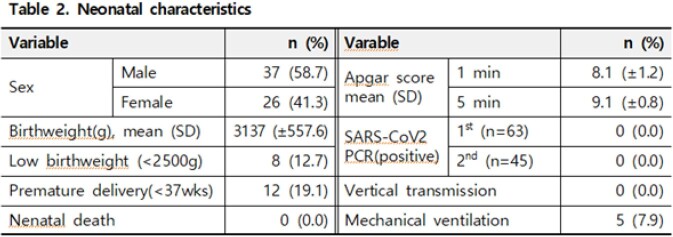

**Conclusion:**

Our experiences about pregnant women with SARS-CoV-2 infection revealed that obstetric outcomes were favorable and the vertical transmission risk was low. Balancing risks about the infection control of pregnant women and their neonates during the COVID-19 pandemic are needed.

**Disclosures:**

**All Authors**: No reported disclosures.

